# Mosquito Behavior and Vertebrate Microbiota Interaction: Implications for Pathogen Transmission

**DOI:** 10.3389/fmicb.2020.573371

**Published:** 2020-12-09

**Authors:** María José Ruiz-López

**Affiliations:** Departamento de Humedales, Estación Biológica de Doñana, Consejo Superior de Investigaciones Científicas, Sevilla, Spain

**Keywords:** microbiota, chemical communication, host preference, mosquitoes, vector-borne disease, pathogen transmission

## Abstract

The microbiota is increasingly recognized for its ability to influence host health and individual fitness through multiple pathways, such as nutrient synthesis, immune system development, and even behavioral processes. Most of these studies though focus on the direct effects microbiota has on its host, but they do not consider possible interactions with other individuals. However, host microbiota can change not only host behavior but also the behavior of other individuals or species toward the host. For example, microbes can have an effect on animal chemistry, influencing animal behaviors mediated by chemical communication, such as mosquito attraction. We know that host skin microbes play a major role in odor production and thus can affect the behavior of mosquitoes leading to differences in attraction to their hosts. Ultimately, the vector feeding preference of mosquitoes conditions the risk of vertebrates of coming into contact with a vector-borne pathogen, affecting its transmission, and thus epidemiology of vector-borne diseases. In this mini review, I provide an overview of the current status of research on the interaction between mosquito behavior and host skin microbiota, both in humans and other vertebrates. I consider as well the factors that influence vertebrate skin microbiota composition, such as sex, genetic makeup, and infection status, and discuss the implications for pathogen transmission.

## Introduction

In a world dominated by microorganisms, animals host diverse microbial communities on different body parts. These communities of symbiotic microorganisms consist of bacteria, archaea, fungi, and viruses, and are of significant importance to host health. In addition to their involvement in important host physiological processes like digestion and nutrient synthesis (Cummings and Macfarlane, 1997), they modulate immune system development (Round and Mazmanian, 2009; Bengmark, 2013) and offer protection against pathogens. This protection is achieved by limiting pathogen adhesion to host cells (Buffie and Pamer, 2013), competing for resources (Kamada et al., 2012), or producing antimicrobial compounds (Fukuda et al., 2011). Changes in composition of the microbiota can lead to physiological changes that will increase the risk of infection by opportunistic pathogens or impair the immune response (Croswell et al., 2009).

The skin is the largest organ in the body and the first barrier of interaction with the environment. The direct effect of skin microbiota on host health has been studied both in humans (Cogen et al., 2008) and wildlife (Williams et al., 2019). For instance, susceptibility to chytridiomycosis in amphibians is associated with differences in skin microbiota composition (Bates et al., 2018). Research also shows that certain skin-associated bacteria inhibit the fungal pathogens that cause chytridiomycosis (Kueneman et al., 2016) and white nose syndrome in bats (Hoyt et al., 2015). In addition, skin microbiota generates odors that act as chemical cues to attract vectors that use olfaction as their main sense to choose their host. This is the case of mosquitoes (Rudolfs, 1922), which are known vectors of many life-threatening diseases, such as malaria, West Nile virus (WNV), or Dengue (Chen et al., 1993; Shin et al., 2002; Balenghien et al., 2008). The transmission of vector-borne diseases depends significantly on how the vector selects its feeding host (Gandon, 2018). Those individuals that are more attractive have a higher risk of coming into contact with a pathogen. Therefore, the specific odor profile of individuals and species that lead to differences in the attraction will profoundly affect the epidemiology of vector-borne diseases. The present paper provides an overview of the current status of research on the interaction between mosquito behavior and skin microbiota both in humans and other vertebrates. The focus of this mini review is mammals and birds because they are the main zoonosis reservoirs. I will discuss what we know about: (i) the role that host skin microbiota plays in odor production; (ii) how changes in skin microbiota composition can lead to differences in mosquito attraction to their hosts both at the intraspecies and interspecies level; and (iii) what factors may affect the skin microbiota composition and thus mediate disease transmission. I will identify both the knowledge gaps and potential future research lines that would help to understand the interaction between mosquito behavior and vertebrate skin microbiota and its impact on health and disease spread.

## The Vertebrate Skin Microbiota and Volatile Production

The microbiota of the human skin is highly complex (Grice et al., 2008), and overall microbial composition varies strongly between individuals depending on factors, such as age, sex, or habits (Fierer et al., 2008). Studies in other mammals have revealed that different species have distinct skin microbial communities, which are in general more diverse than the microbial communities in humans (Council et al., 2016; Ross et al., 2018). Some of these differences were driven by changes in the abundance of certain groups of bacteria (A decrease in *Actinobacteria* and an increase of *Chloroflexi* and *Bacteroidetes*; Ross et al., 2018). For some mammals, including humans, skin sites vary in their microbial composition and skin microorganisms tend to be more abundant around glands and skin pouches (Verhulst et al., 2010a; Leclaire et al., 2014; Kamus et al., 2018).

Studies in birds show that like mammal skin, feathers harbor their own microbial community with a composition that varies between individuals and species (Engel et al., 2018). *Staphilococcus*, *Bacillus*, *Pseudomonas*, and *Stenotrophomona* are some of the most common bacteria genera found on the plumage of different bird species (Whittaker et al., 2019). Birds’ main holocrine gland is the uropygial gland, which produces an oily secretion that birds preen onto their feathers. A significant amount of this secretion is formed by lipids that may control the growth of feather-degrading bacteria living on the plumage or be used as nutrients by feather microbiota that produce body odors (Purton, 1986). In addition, it has been shown in several species that uropygial gland area and secretion produced also contain bacteria (Martín-Vivaldi et al., 2010; Whittaker et al., 2019).

Symbiotic microbial communities in the skin of both mammals and birds play a significant role in odor production through the generation of volatile compounds like linear alcohols, methyl ketones, and carboxylic acids (Madigan et al., 2010). Different species of skin bacteria have distinct metabolic routes that produce a variety of compounds. For example, in humans, the odor associated with the axillary glands has been linked to *Corynebacteria* (Leyden et al., 1981), which generate volatile fatty acids (James et al., 2004). *Staphylococcus* species, common in humans and birds, can metabolize branched-chain amino acids into short-chain amino acids that are volatile and highly odorous (James et al., 2004). In birds, volatile compounds from the uropygial gland secretion were linked with several bacteria genera, including *Pseudomonas* and *Staphylococcus* (Whittaker et al., 2019). Therefore, skin/feather microbiota that differs not only in composition but also in the abundance of certain bacteria species will generate a characteristic odor profile for each individual (Theis et al., 2012; Leclaire et al., 2014). These olfactory cues are associated with host sex, age, and social interactions, indicating the potential for chemical communication (Fierer et al., 2008; Theis et al., 2012; Leclaire et al., 2014).

## Mosquito Behavioral Response to Skin Microbiota Volatiles in Vertebrates

**Figure 1:**
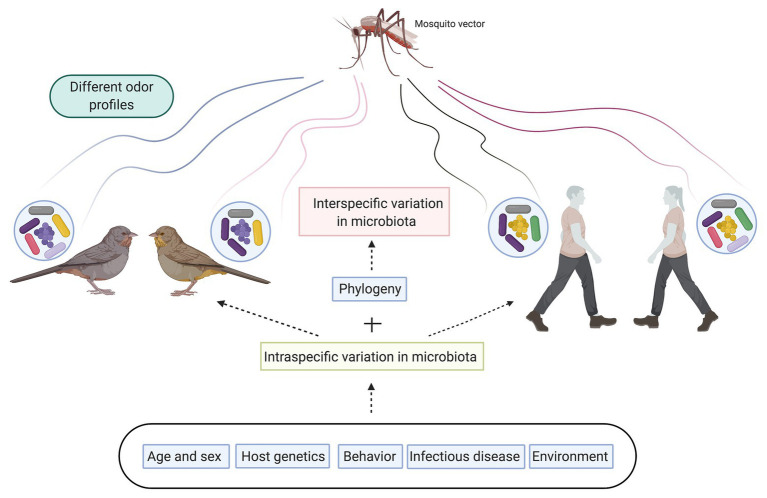
Graphical outline of factors affecting skin and plumage microbiota composition leading to intraspecific and interspecific differences in microbiota profiles. These different microbiota profiles will produce distinct odor profiles that mosquitos use as cue to select host they will feed on.

The idea that the volatile compounds produced by skin microbiota could attract mosquitoes was first proposed by Schreck and James (1968). They tested whether the volatiles of a broth where they had grown bacteria (*Bacillus cereus*) was attractive to *Aedes aegypti*. The results showed that the volatiles produced were attractive. Since then, several studies have shown that human skin bacteria produce volatiles that are attractive to mosquitoes (De Jong and Knols, 1995; Braks et al., 2000; Verhulst et al., 2009). These studies confirm that skin microbiota influences host seeking behavior in mosquitoes. But skin microbiota composition is unique for each individual, contributing to the generation of distinct odor profiles, which drive mosquito host seeking behavior and selection (Verhulst et al., 2018; Figure 1). In fact, it is clear that mosquitoes show a preference to bite certain individuals, demonstrating that there are intraspecific differences in the attractiveness of individuals to mosquitoes (see Bernier et al., 2002 and Mukabana et al., 2002 for examples in humans). Several studies in humans have shown that these differences in attractiveness are mediated by the microbiota. Two species of bacteria were identified as key in determining attractiveness, *Pseudomonas aeruginosa*, and *Staphilococcus epidermis*. Mosquito species of the *Anopheles gambiae* complex were not attracted to the odor produced by *P. aeruginosa*, while the odor of *S. epidermis* was attractive (Verhulst et al., 2010b, 2011). In addition, Verhulst et al. (2011) demonstrated that the more attractive individuals had less diverse microbiota, but hosted a higher abundance of some bacteria including *Staphylococcus*. In contrast, *Pseudomonas* was more abundant in poorly attractive individuals. This pattern of intraspecific differences in attractiveness to mosquitoes plays a significant role in pathogen exposure and pathogen transmission dynamics. The more attractive individuals have more likelihood of being bitten, increasing their chances of being infected by mosquito-borne pathogens and driving pathogen transmission.

Although most studies linking bacteria, volatile production and mosquito attraction have been carried out in humans and anthropophilic mosquitoes, studies in other species show similar patterns. For example, in some species of birds, both the uropygial gland secretion and volatile compounds of skin and plumage have been shown to attract mosquitoes (Russell and Hunter, 2005; Bernier et al., 2008; but see Douglas et al., 2005). However, despite the evidence that bacteria in the uropygial gland and plumage produce volatile compounds that can be used in olfactory communication (Maraci et al., 2018), there is no study yet linking the production of volatile compounds that specifically attract mosquitos with bacteria species in birds.

Mosquito species differ widely in their host preference, defined as “the trait to preferentially select certain host species above others” (Takken and Verhulst, 2013). While many mosquito species are opportunistic and their host preference is mainly driven by host abundance and availability, other species show strong preferences, feeding mainly on mammals (mammophilic), birds (ornithophilic), or other vertebrate groups such as reptiles (Chaves et al., 2010; Martínez-de la Puente et al., 2015). Some mosquito species even exhibit a marked preference to feed on a single host species, no matter the circumstances, like *An. gambiae* sensu stricto that is highly anthropophilic (Pates, 2002). Host preference is one of the key determinants of the vectorial competence for disease transmission because it determines the risk that a vertebrate species will come into contact with a pathogen (Gandon, 2018). Those species of mosquitoes with plastic behavior that adapt to feed on available vertebrate species play a key role in pathogen transmission between species and may drive zoonosis emergence (Takken and Verhulst, 2013; Yakob et al., 2018). One of the classic examples illustrating this situation is the transmission of WNV to humans. This virus is maintained in nature in an enzootic cycle involving ornithophilic mosquitoes, which are the transmission vector. *Culex pipiens* is one of the primary vectors of WNV and typically feeds on birds. However, under certain circumstances like migration events when the abundance of birds changes, they switch host to humans contributing to the spread of WNV in humans (Farajollahi et al., 2011). To further understand host selection in mosquitoes that act as vectors of WNV, Spanoudis et al. (2020) compared the responses of *Culex quinquefasciatus* and *Cx. pipiens molestus* to volatiles of different bird species. *Culex quinquefasciatus* shows phenotypic plasticity and its feeding preference varies in different ecotypes (Mboera and Takken, 1999; Molaei etal., 2006). *Culex pipiens molestus* is an anthropophilic form of *Cx. pipiens* (Vinogradova, 2000) adapted to urban environments. Both species of mosquitoes responded to bird volatiles but differed in their preference (Spanoudis et al., 2020). *Culex quinquefasciatus* responded to the volatiles of both sexes of chickens (*Gallus Gallus domesticus*), and female pigeons (*Columba livia*). *Culex pipiens molestus* responded to the volatiles of chickens and magpies (*Pica pica*). These differences in feeding preference are critical for disease transmission. Out of the species included in the study, magpies are the most susceptible to WNV (Jiménez de Oya et al., 2018) allowing its circulation in wild populations (Napp et al., 2019). Mosquitoes that prefer feeding on both magpies and humans facilitate transmission of WNV between the bird reservoir and humans. This example shows that to determine the potential of mosquitoes as disease vectors it is crucial to understand their feeding preferences, which involves studying how mosquitoes select their host, and how odor profiles and microbiota are influencing this choice. In this sense, several studies in different taxa have analyzed if the odor of different species was equally attractive to a set of species of mosquitoes. Bakker et al. (2020) set traps with odors from chimpanzees (*Pan troglodytes*), humans, and cows (*Bos taurus*) and identified the mosquitoes captured. Most of the mosquito species trapped during this study were equally attracted to all mammal species tested showing a generalistic host preference. Another study carried out with house sparrows (*Passer domesticus*) showed no differences in the response of the ornitophilic mosquitoes (*Cx. pipiens*) and mammophilic mosquitoes [*Aedes* (*Ochlerotatus*) *caspius*] when exposed to the uropygial gland secretions (Díez-Fernández et al., 2020a). Therefore, it seems that the preference of ornithophilic mosquitoes for avian hosts is not associated with attraction to the uropygial gland secretion odor. However, it is possible that mosquitoes respond to specific volatile compound released when the secretion is deposited on the feathers and metabolized by the bacterial community (Díez-Fernández et al., 2020a). In humans, it has been shown that this variation in mosquito response is in fact mediated by the composition of skin microbiota volatiles (Busula et al., 2017). In this study, two species of mosquitoes with different host preferences (*An. gambiae* and *An. arabiensis*), exhibited a different response to the volatiles released from skin bacteria from three different mammal species (human, cow, and chicken). *Anopheles gambiae* showed higher attraction to bacteria volatiles of human origin, and displayed a specialized response to four species of bacteria preferring volatiles from *Corinebacterium minutissimum*, one of the most abundant microbes in human skin. In contrast, *An. arabiensis* showed more attraction to bacterial volatiles from chickens responding equally to all species of bacteria tested. More studies like this one, including different vertebrate and mosquito species, will help to understand how skin microbiota drives interspecies differences in mosquito attraction and mediates potential pathogen transmission.

## Factors Affecting Skin Microbiota Composition: Potential Impact on Mosquito Attraction and Pathogen Transmission

Skin microbiota composition is influenced by multiple factors. In addition to the phylogeny of the host discussed above, age, sex, behavior, genetic makeup of the host, and environmental variables, also impact skin microbiota composition (Fierer et al., 2008; Theis et al., 2012; Leclaire et al., 2014; Figure 1). Therefore, these factors will indirectly affect how mosquitoes select their host leading to differential exposure to vector-borne pathogens among individuals. Some studies have analyzed the effect of sex on attractiveness to mosquitoes. One study found no differences in exposure from males and females of great tit nestlings (*Parus major*) to bites of *Cx. pipiens* (Cozzarolo et al., 2019). In contrast, Spanoudis et al. (2020) showed that female pigeons were attractive to *Cx. quinquefasciatus* while males were not attractive. Regarding genetic makeup, it is recognized that there is a genetic component determining susceptibility to being bitten (Fernández-Grandon et al., 2015) and odor profile is partially genetically based (Kuhn and Natsch, 2009). Interestingly, the genes of the major histocompatibility complex (MHC), the most important cluster of immune genes, are also some of the most important candidate genes for explaining body odor (Penn and Potts, 1998) and have been linked in humans with differences in mosquito attraction (Verhulst et al., 2013). Two combined hypotheses explain the potential effect of MHC on odor. First, it has been proposed that degraded MHC molecules directly influence odor (Pearse-Pratt et al., 1992). Second, it is known that MHC variation correlates with gut microbiota composition and diversity (Bolnick et al., 2014), and that skin microbiota triggers immune responses associated with the MHC (Dillon et al., 2000). Furthermore, in two seabird species, there is an association between MHC genotypes and microbiota composition in the uropygial gland (Pearce et al., 2017) and plumage (Leclaire et al., 2019). Thus, MHC genes may also influence the odor of individuals by shaping the microbiota composition of skin/feathers and adjacent glands, leading to differences in exposure to vector-borne pathogens among individuals.

A factor that can impact microbiota composition and has not been studied so far is infectious disease. It is known that mosquito-borne pathogens, like malaria (Taniguchi et al., 2015), are associated with changes in gut microbiota. Little is known however, about potential changes in skin microbiota. But changes in skin microbiota composition might be critical in determining disease transmission, if the new profiles make the infected individuals more attractive to mosquitoes. Although so far there are no studies associating changes in skin microbiota due to infection with attractiveness to mosquitoes, research in several vertebrate species shows that individuals infected with *Plasmodium* spp. are more attractive to mosquitoes [rodents (Ferguson and Read, 2004; De Moraes et al., 2014), canaries (*Serinus canaria*; Cornet et al., 2013a,b), house sparrows (Díez-Fernández et al., 2020b), and humans (Lacroix et al., 2005; Batista et al., 2014; Robinson et al., 2018)]. These studies show that pathogen infection, in this case malaria, can change an individual’s odor profile, making the infected individuals more attractive to mosquitoes. If infected individuals are then more frequently bitten, this would enhance the transmission of the pathogen in the population. Although all of these studies identify odor profiles, it seems reasonable to think that potential changes in skin microbiota composition may play a substantial role and it should be studied if the skin and plumage microbiota also change during different infections caused by mosquito-borne pathogens.

## Conclusion and Future Directions

Vector-borne pathogens are the causative agents of some of the most harmful diseases in humans, like malaria, dengue, and yellow fever. In addition, they are also the cause of enormous economic losses in domestic animals and population declines among wildlife. Understanding how vectors select their feeding host is critical to understand parasite transmission. For some vectors, like mosquitoes, odor is the main cue to select their hosts. Multiple studies confirm that the microbiota on skin and plumage plays a critical role in body odor production. Therefore, the differences in attractiveness of individuals and species to mosquitoes may be explained by variation in the microbiota composition. However, most of these studies are associative and do not demonstrate causal relationships. One of the challenges is linking bacteria species with volatiles and odor production that will cause differences in the attractiveness to mosquitoes. To bridge this gap, a potential avenue would be to stablish gnotobiotic animal models colonized by known microbes. These animal models in combination with the use of “omic” tools, like metatranscriptomics and metabolomics, will help to link bacteria species with metabolic pathways that are responsible for the volatile by-products. In addition, to further comprehend the mechanisms underlying the interaction between mosquito behavior and host microbiota, more studies including different species of vertebrates and mosquitoes are necessary. This will help to understand which are the cues generalists and specialized mosquitoes are responding to and what are the common patterns of host-seeking behavior. For example, mosquitoes with different host feeding preferences may be responding to different odor profiles generated by different microbiota composition or specific bacteria. In contrast, mosquitoes with a generalist feeding preference might be responding to common odor profiles across species generated by bacteria that are found across different host species. It will also be important to gain a better understanding of how the factors that affect microbiota affect host selection by mosquitoes. Some examples of what can be done include looking at the genetic basis of skin microbiota composition (Davenport, 2016) using genome wide association studies, or linking infectious disease caused by mosquito-borne pathogens with changes in skin microbiota that can lead to differences in attractiveness to mosquitoes. Overall, future research should include a combination of laboratory and ecological studies on the interaction between skin microbiota and host seeking behavior of mosquitoes that will help to reveal some of the most important factors underlying pathogen amplification and transmission.

## Author Contributions

MR-L designed the mini review and wrote the manuscript.

### Conflict of Interest

The author declares that the research was conducted in the absence of any commercial or financial relationships that could be construed as a potential conflict of interest.
